# A Case Report of Promising Response to Combination Therapy With an Immune Checkpoint Inhibitor (Pembrolizumab) and Multi‐Targeted Tyrosine Kinase Inhibitor (Pazopanib) in Metastatic De‐Differentiated Chondrosarcoma

**DOI:** 10.1002/cnr2.70426

**Published:** 2026-01-29

**Authors:** Matthew Youssef, Peter Grimison

**Affiliations:** ^1^ Royal Prince Alfred Hospital Sydney New South Wales Australia; ^2^ Chris O'Brien Lifehouse Sydney New South Wales Australia; ^3^ University of Sydney Sydney New South Wales Australia

## Abstract

**Background:**

We present the case of 72–year‐old male with metastatic de‐differentiated chondrosarcoma (“DDCS”). DDCS is a rare soft tissue cancer that carries a dismal prognosis in the metastatic stage, and is resistant to both traditional chemotherapy and radiotherapy. There is a distinct lack of proven systemic therapies.

**Case:**

This case report is distinguished in that our patient had a significant clinical response to combination therapy with an immune checkpoint inhibitor (Pembrolizumab) and a multi‐targeted tyrosine kinase inhibitor (Pazopanib). Our patient presented with bony pain after suffering a pathological fracture of the left humerus after grabbing a fence. He did not report prior constitutional symptoms or bony pain. On examination he was clinically in atrial fibrillation and reported reduced exercise tolerance with a New York Heart association grading of 2. There were no other pertinent clinical findings. FDG PET‐CT scan revealed a 10cmx5cm destructive intra‐osseous lesion of the proximal humerus, markedly FDG‐avid, without evidence of metastatic disease. He underwent immediate surgical resection, followed by adjuvant radiotherapy to the left humerus. Tumour histology revealed a high‐grade DDCS. Genetic sequencing revealed alterations in IDH2, PTCH1 and TERT promoter. Restaging FDG PET scan 3 months after diagnosis revealed lung metastases. The patient was commenced on Vismodegib with best disease response of progressive disease. Eight months after diagnosis he was commenced on combination therapy of Pazopanib and Pembrolizumab, with significant reduction in size of lung metastases. He sustained a progression free period of 6 months on this regime. Treatment course was complicated primarily by hepatotoxicity, which resolved with dose reduction of Pazopanib. Eleven months after diagnosis, FDG PET‐CT revealed relapse and significant progression of metastatic disease and systemic therapy was ceased. The patient passed away 14 months after diagnosis.

**Conclusion:**

This case report is a valuable example of promising emerging systemic therapies for advanced DDCS, where the present standard of care lacks a repertoire of effective therapies.

## 1 Introduction

Dedifferentiated chondrosarcomas (“DDCS”) are rare sarcomas of bone that carry a dismal prognosis [1]. A retrospective multi‐centre study of 337 patients found that patients with metastatic disease at diagnosis had a median survival of 5 months and survival rate of 10% at 2 years, and patients with localised disease at diagnosis had a survival rate of 28% at 10 years [2].

DDCS are typically chemo‐resistant, and chemotherapy very rarely provides a durable response [3]. The current standard of care involves regimes based on a combination of doxorubicin with cisplatin or ifosfamide, with consideration of use of IDH‐1 inhibitors, given the high proportion of tumours with IDH 1/2 mutations [4]. Newer targeted cancer therapies, such as immune checkpoint inhibitors and tyrosine kinase inhibitors, as was used in this case, have not been formally evaluated for DDCS, and are isolated to case reports involving Pembrolizumab [5, 6], a phase 1/2 non‐randomised trial that included 8 patients with DDCS treated with Pembrolizumab [7], an a case series that included one patient with DDCS and prolonged disease stabilisation on Pazopanib [8].

## 2 Case

In August 2022, a 72 year old man with a past history of ischaemic heart disease, atrial fibrillation and gastro‐oesophageal reflux presented to a community hospital with a pathological fracture of the left humerus after grabbing a fence. He did not report prior constitutional symptoms or bony pain. On examination he was clinically in atrial fibrillation and reported reduced exercise tolerance with a New York Heart association grading of 2. There were no other pertinent clinical findings.

Imaging revealed a 10 cm × 5cm destructive intra‐osseous lesion in the proximal humerus which was largely lytic but with focal calcification, very T2 hyperintense on MRI and markedly FDG‐avid. No metastases were identified. He underwent surgical resection of the humerus lesion with clear margins, followed by adjuvant radiotherapy to the left humerus.

Histology revealed a high‐grade dedifferentiated chondrosarcoma, with background conventional chondrosarcoma and enchondroma. There were multiple areas of invasion and extension into the cortex and soft tissue, and venous invasion. Genetic sequencing of the tumour revealed alterations in IDH2, PTCH1 and TERT promoter. Surveillance FDG PET‐CT scan 3 months after resection of the primary revealed widespread but asymptomatic lung metastases. He was referred to Chris O'Brien Lifehouse in Sydney for further evaluation and consideration of systemic medical therapy.

## 3 Treatment

**FIGURE 1:**
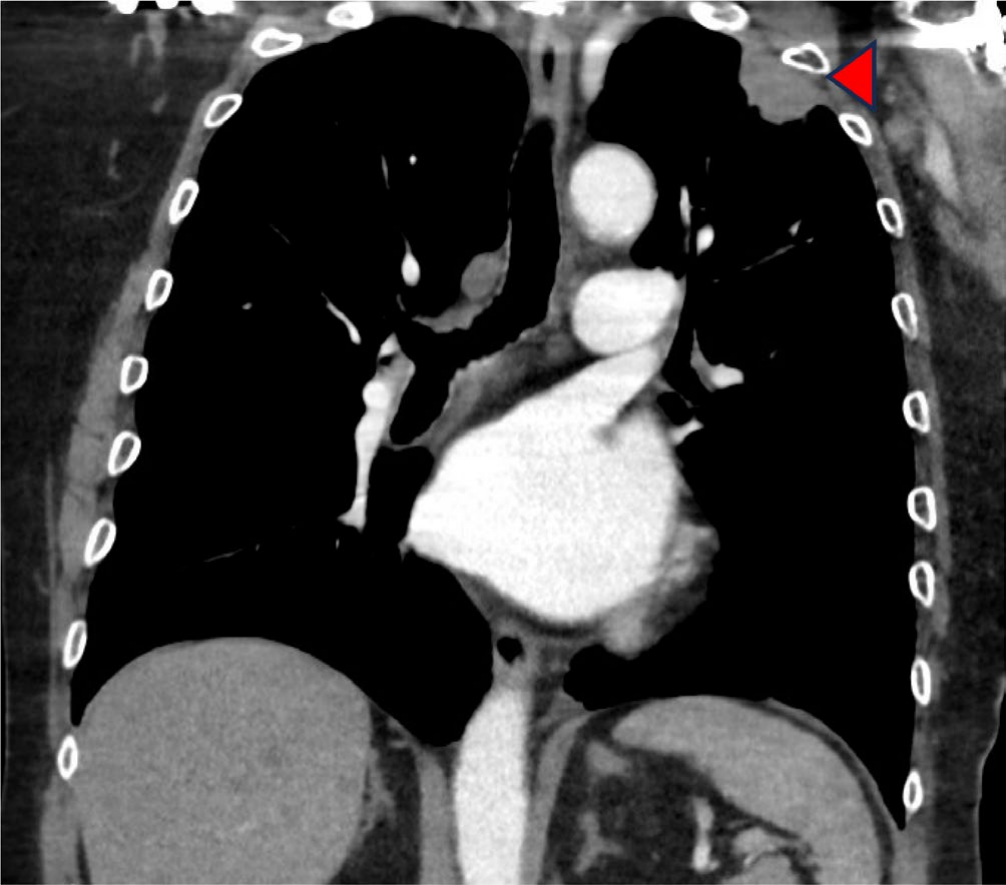
FDG PET‐CT scan showing pulmonary metastasis in the apex of the left upper lobe (see arrow) prior to treatment with pembrolizumab and pazopanib.

**FIGURE 2:**
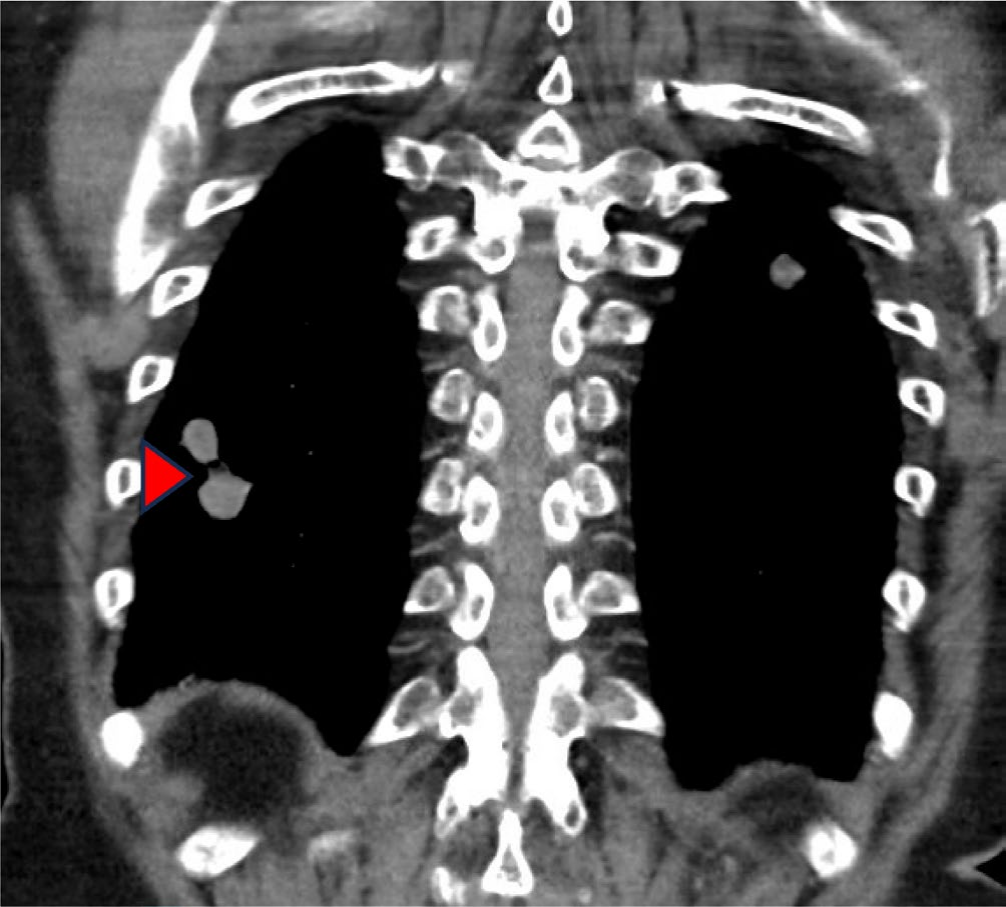
FDG PET‐CT scan showing pulmonary metastases along the oblique fissure in the right lobe (see arrow) prior to treatment with pembrolizumab and pazopanib.

The patient declined chemotherapy and enrolled in a clinical trial for patients with PTCH1 mutations of an oral Hedgehog pathway inhibitor (Vismodegib 150 mg daily orally), however progress FDG PET‐CT scan after 2 months of therapy revealed progression of disease, most notably pulmonary metastases in the apex of the left upper lobe (Figure 1) and along the right oblique fissure (Figure 2) and Vismodegib was ceased.

**FIGURE 3:**
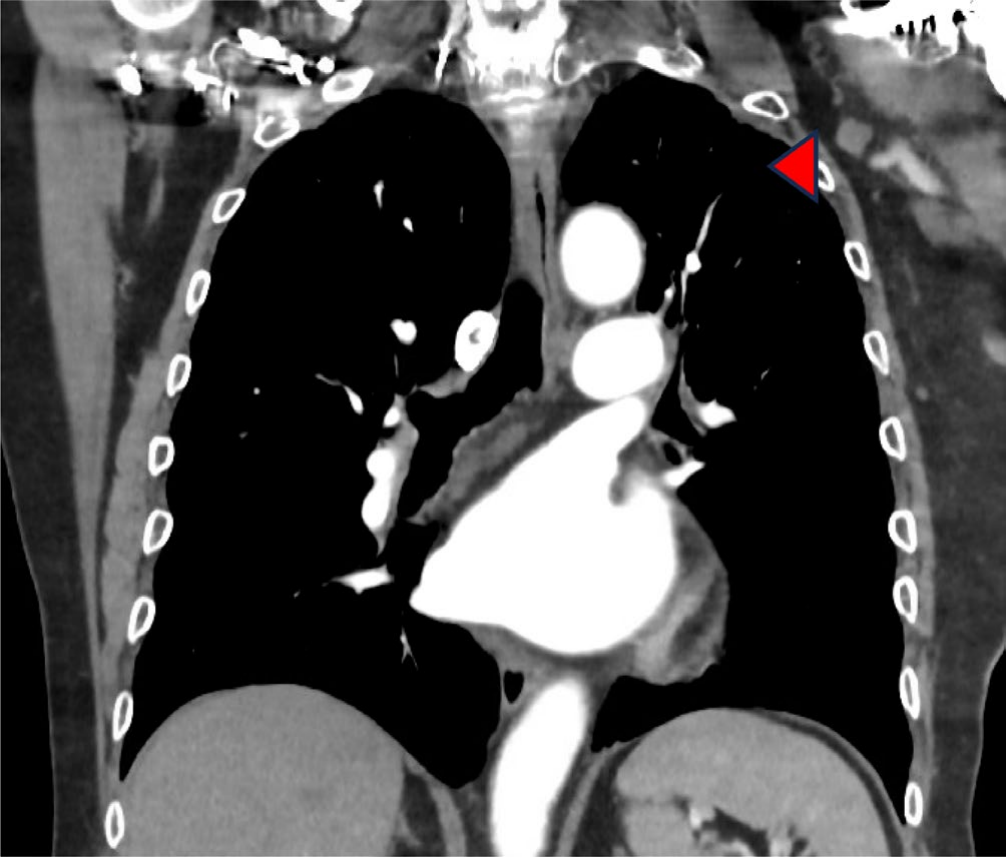
FDG PET‐CT scan showing significant resolution in metastatic lesion (see arrow) in the apical region of the left upper lobe seen in Figure 1 following treatment with pembrolizumab and pazopanib.

**FIGURE 4:**
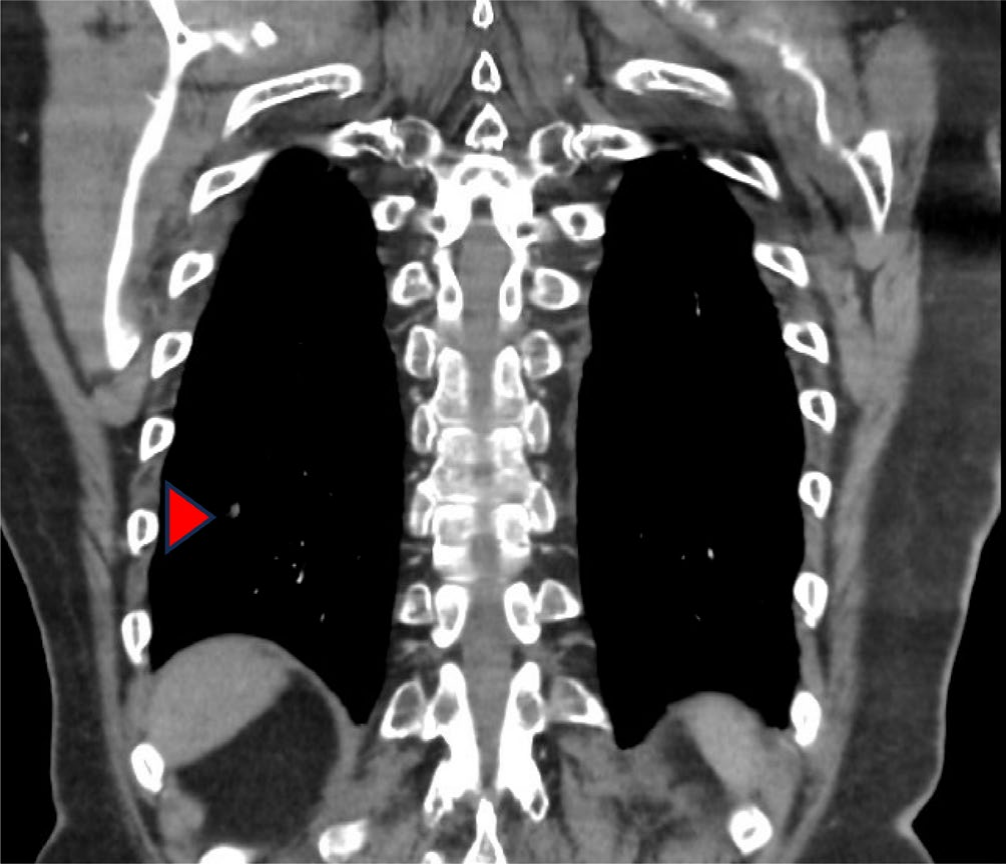
FDG PET‐CT scan showing significant resolution of 2× metastatic lesions along the oblique fissure (see arrow) of the right lobe seen in Figure 2 following treatment with pembrolizumab and pazopanib.

Approximately 6 months after diagnosis, he commenced second‐line systemic therapy with concurrent pembrolizumab IV 200 mg q3 weekly and pazopanib 800 mg daily. After 2 months of therapy, restaging FDG PET‐CT revealed significant reduction in lung metastases (Figures 3 and 4), which was maintained after 4 months of therapy on further FDG PET‐CT scan. During this period, pembrolizumab was intermittently withheld for Grade 1 asymptomatic hepatotoxicity; and Pazopanib was intermittently withheld for Grade 2 hepatotoxicity, hypertension and Grade 3 fatigue and ataxia. Hepatotoxicity improved with dose reduction of pazopanib to 400 mg daily and rechallenge of pembrolizumab. After 6 months of therapy, FDG PET‐CT showed significant progression of disease at the primary site, mediastinal lymph nodes, lungs and bone, and treatment was ceased.

## 4 Outcome and Follow‐Up

In the context of declining performance status, the patient declined third‐line systemic therapy with an IDH2 inhibitor or chemotherapy. One‐month later, the patient presented to hospital with acute anterior chest wall pain and dyspnoea due to pathological rib fractures and a malignant pleural effusion. The decision was made to cease systemic therapy at this time in favour of full palliative management. The patient passed away 14 months after diagnosis. The timeline of clinical events is outlined in Table 1.

**TABLE 1:** Timeline of clinical events.

Date	Clinical event
August 2022	Patient suffers pathological fracture of left humerus, after a fall on outsretched arm.
	Underwent MRI left shoulder—10 cm × 5 cm intra‐osseous, lytic lesion in proximal humerus.
	Underwent FDG PET‐CT with nil evidence of metastases.
	Underwent surgical resection of left humeral lesion. Histology revealing high‐grade dedifferentiated chondrosarcoma. Invasion and extension into cortex, soft tissue and venous.
October 2022	Next generation sequencing of primary tumour lesion showing alterations in IDH2, PTCH1, TERT promotor genes.
November 2022	Surveillance FDG PET‐CT showing widespread pulmonary metastases.
December 2022–February 2023	Vismodegib administration.
February 2023	Surveillance FDG PET‐CT showing disease progression with new pulmonary metastases (see Figures 1 and 2). Vismodegib therapy ceased.
March–July 2023	Received pembrolizumab and pazopanib. Restaging FDG PET‐CT in May 2023 revealed significant reduction in lung metastases (Figures 3 and 4). Restaging FDG PET‐CT in July 2023 revealed widespread disease progression. Systemic therapy ceased in July 2023 with transition to best supportive care.
September 2023	Presented to hospital for palliation with anterior chest wall pain secondary to pathological rib fractures and malignant pleural effusion.
	Palliative radiotherapy for rib metastases.
October 2023	Deceased.

## 5 Discussion

The average age of presentation of DDCS is in the fifth decade of life [1]. The majority of primary lesions occur in medullary bone (most common sites being the pelvis, femur and humerus) [9]. For non‐metastatic disease, surgery to the primary site is considered the most effective treatment as it has a clear survival benefit, particularly where clear margins are achieved [10]. Limb salvage is more commonly performed over amputations as there is no established mortality benefit for the latter [11]. Patients with primary lesions of the axial skeleton achieve adequate surgical margins less frequently than those with appendicular skeletal lesions. Unfortunately the rate of progression to metastatic disease has been reported at over 50% in some studies [12], and there is no proven role for adjuvant systemic therapy.

Contemporary international guidelines regarding systemic therapy for DDCS recommend consideration of neoadjuvant or adjuvant chemotherapy for localised disease using a regimen designed for osteosarcoma [13], or consideration of IDH1 inhibitors in the setting of a potentially susceptible IDH1 mutations [4], albeit based on very low‐level evidence.

### 5.1 Systemic Chemotherapy

There is conflicting data regarding the efficacy of systemic chemotherapy in DDCS, which typically involves regimens established for osteosarcoma based on a combination of doxorubicin and cisplatin or ifosfamide. Some studies found modest benefit in patients treated with primary surgery followed by adjuvant ifosfamide [14], as well as adjuvant doxorubicin and cisplatin [15], however other studies suggest no survival benefit, particularly in cases of metastatic or unresectable disease, high‐grade dedifferentiated tumours and patients with severe co‐morbidities [16]. A retrospective study conducted by the European Musculo‐Skeletal Oncology Society (EMSOS) with 337 patients with DDCS showed no statistically significant improvement in the survival of patients receiving systemic chemotherapy. In this study, the majority of patients were under 60 years old, had no metastases and had undergone limb salvage surgery. The presence of a pathological fracture was the most significant poor prognostic factor [16].

The EURO‐BOSS clinical trial, which included 57 patients with DDCS (34 of which had only localised disease) reported a 5‐year overall survival of 39% with a regime of doxorubicin, ifosfamide and cisplatin (and post‐operative methotrexate that was added in cases of poor histological response) [17]. This was higher than previous retrospective studies and previous clinical trials that report ranges between 10% and 24% [17]. A retrospective study containing 42 patients with DDCS were treated with doxorubicin and an alternative agent (most commonly ifosfamide). Approximately 20% of the patients in that study had an objective clinical response [18].

### 5.2 IDH Inhibitors

Although IDH inhibitors are recommended in the recent NCCN guidelines for DDCS with IDH mutations [4], evidence for benefit is lacking, and their role has not been well established. IDH1/2 mutations are found in over 50% of DDCS [19]. A phase 1 study of the IDH‐1 mutant inhibitor ivosedinib for IDH1 mutant advanced solid tumours included 6 patients with DDCS, for whom there were no objective responses and only 30% progression‐free survival at 3 months [19]. Further trials evaluating their use are ongoing.

### 5.3 Immune Checkpoint Inhibitors

There is limited data regarding the role of immune checkpoint inhibitors for DDCS. A recent case report by Cohen‐Nowak et al. described a 44 year‐old female with chondrosarcoma who achieved a sustained response to Pembrolizumab [6]. She presented with a Stage IIA primary chondrosarcoma of the right scapula. At the time of commencing Pembrolizumab she had pulmonary metastases and marked dyspnoea. There was an immediate response with significant improvement in dyspnoea, as well as significant reduction in pulmonary metastases and lymph node size.

Strong PD‐L1 expression in DDCS suggests that immunotherapy may be a potential therapeutic approach. A recent analysis of chondrosarcomas found that 9 of 22 (41%) of DDCS tumours expressed PD‐L1 positivity in at least 50% of cells, raising the hypothesis of sensitivity to immunotherapy [20]. SARC 028, a multi‐centre, open‐label phase 2 clinical trial evaluating pembrolizumab (which is an anti‐PD1 immune checkpoint inhibitor) for advanced soft tissue or bone sarcomas reported that one of the five patients (20%) with chondrosarcoma achieved an objective response [21]. A phase 1/2 non‐randomised trial of 37 patients with advanced sarcoma evaluating the combination of doxorubicin chemotherapy with pembrolizumab reported that 3 out of the 8 patients (38%) with chondrosarcoma achieved a tumour response [7].

One patient with DDCS receiving nivolumab alone in a retrospective study (*n* = 28) of nivolumab with or without Pazopanib for metastatic or unresectable sarcoma had a partial response [22]. In a retrospective review of the medical records from five sarcoma centres in the United States, 74 patients with DDCS were identified with a median age of 63 years [8]. 5 were treated with immune checkpoint inhibitors. The best response was a partial response to pembrolizumab that was ongoing at the time of date cut‐off. This patient had a PD‐L1 tumour proportion score of 7%. Two of the patients identified had prolonged stable disease. One who had received ipilimumab/nivolumab had stable disease for 24 weeks. The other patient who received pembrolizumab monotherapy had stable disease for 48 weeks [8].

In 2022, Singh et al. published a case report which described a 70 year old male with a primary DDCS lesion of the right proximal femur, who was treated with palliative resection. He subsequently developed metastatic disease and achieved a sustained complete response to pembrolizumab for 24 months [5].

### 5.4 Tyrosine‐Kinase Inhibitors

The role of multi‐targeted tyrosine kinase inhibitors in DDCS has not been established. A phase 2 study of pazopanib in 47 patients with unresectable or metastatic chondrosarcomas reported a partial response rate of 2% and disease control rate of 43% at 16 weeks, however excluded patients with DDCS [23]. A randomised phase 2 study of regorafenib or placebo for advanced chondrosarcoma reported a response rate of 8% and progression‐free survival of 61% at 12 weeks, however did not report on responses for the 2 patients with DDCS [24].

Preliminary results from a phase I/II study of sunitinib and nivolumab in advanced soft tissue and bone sarcoma (IMMUNOSARC, *n* = 40) included 4 patients with DDCS, of which 1 of 4 patients (25%) achieved a complete response that was ongoing at 22 months [25]. Recruitment is ongoing to the DDCS cohort (*n* = 23) [26], which would consider a 6‐month progression‐free survival rate of 70% to be worthy of further evaluation.

## 6 Conclusion

De‐differentiated chondrosarcoma is a rare soft tissue tumour that classically responds very poorly to traditional chemotherapy and radiotherapy, and thus carries a very poor prognosis. Current guidelines for this type of cancer do not carry robust evidence, and further research into alternative treatment options are needed. This case report is unique in its demonstration of an effective clinical response with novel systemic therapies that differentiates from the standard of care. Newer targeted therapies including PDL‐1 inhibitors and multi‐targeted tyrosine kinase inhibitors provide some hope that clinical outcomes may improve for this disease in future, and should be definitively evaluated in prospective clinical trials.

## Author Contributions

Conceptualization: P.G. and M.Y. Methodology: P.G. and M.Y. Investigation: M.Y. Formal analysis: P.G. and M.Y. Resources: P.G. and M.Y. Writing – original draft: P.G. and M.Y. Writing – review and editing: P.G. and M.Y. Visualization: P.G. and M.Y. Supervision: P.G. All authors had full access to the data in the study and take responsibility for the integrity of the data and the accuracy of the data analysis.

## Funding

The authors have nothing to report.

## Ethics Statement

The research leading to this case report has been approved by the Sydney Local Health District Human Research Ethics Committee (SLHD HREC), under a waiver of consent for sarcoma patients at our institution (Chris O'Brien Lifehouse). The protocol number is X19‐0246 and ethics approval number 2019/ETH1837.

## Consent

The patient has signed an explicit written consent for anonymized patient information to be published in this article, including case details and use of images.

## Conflicts of Interest

The authors declare no conflicts of interest.

## Data Availability

The data that support the findings of this study are available on request from the corresponding author. The data are not publicly available due to privacy or ethical restrictions.
